# First record of a new microsporidium pathogenic to *Gonipterus platensis* in Brazil

**DOI:** 10.1038/s41598-021-90041-9

**Published:** 2021-05-26

**Authors:** Carolina Jordan, Vanessa Rafaela de Carvalho, Gabriel Moura Mascarin, Leiliane Rodrigues dos Santos Oliveira, Christopher A. Dunlap, Carlos Frederico Wilcken

**Affiliations:** 1 School of Agricultural Sciences, São Paulo State University (UNESP), Campus of Botucatu, Av. Universitária, 3780, Altos do Paraíso, Fazenda Experimental Lageado, Botucatu, SP 18610-034 Brazil; 2 Laboratory of Environmental Microbiology, Brazilian Agricultural Research Corporation, Embrapa Environment, Rodovia SP-340, km 127.5, Jaguariúna, SP 13918-110 Brazil; 3 Botucatu Medical School, Dept. Internal Medicine, Sao Paulo State University (UNESP), Campus of Botucatu, Av. Prof. Mário Rubens Guimarães Montenegro, s/n, Botucatu, SP 18618-687 Brazil; 4 USDA, Agricultural Research Service, National Center for Agricultural Utilization Research, Crop Bioprotection Research Unit, 1815, N. University St, Peoria, IL 61604 USA

**Keywords:** Ecology, Microbiology

## Abstract

Microsporidia are naturally occurring fungal-related parasites that can infect nearly all animal hosts, but their biocontrol potential of insect pests is routinely overlooked in agriculture and forestry. This research brings the first report describing the natural occurrence of a microsporidium causing disease in field-collected populations of the invasive eucalyptus snout beetle, *Gonipterus platensis* (Coleoptera: Curculionidae), a major destructive pest of eucalyptus plantations in Brazil. Adult beetles were collected during field surveys in commercial eucalyptus plantations in southern Brazil to be examined and dissected with typical symptoms to verify presence of microsporidian spores in haemolymph. From 14 plantations in different sites, the natural infection occurrence in these populations ranged from 0 to 65%, while a lab colony exhibited an infection incidence of 70%. Spore density in haemolymph of symptomatic insects averaged 2.1 (± 0.4) × 10^7^ spores/beetle. Symptoms in infected adults were identified by an abnormal abdomen with malformation of the second pair of wings, impairing their flight activity. Electron transmission microscopy of the pathogen showed morphological features similar to species belonging to the genus *Nosema* or *Vairimorpha.* Phylogenetic analysis of the full-length small subunit ribosomal RNA gene suggests this pathogen’s placement in the genus *Vairimorpha,* but with a sequence identity of ~ 94% with the nearest neighbours. The low level of sequence identity suggests this pathogen may represent a novel taxon in the genus and further requires whole genome sequencing for definitive taxonomic resolution. These findings provide insights on the natural occurrence of this novel pathogen of this invasive pest in *Eucalyptus* plantations in Brazil. Further studies are needed to determine potential of this microsporidium in the design of conservative or augmentative biological control programs for this invasive pest.

## Introduction

Various environmental, biological and genetic factors can influence performance and fitness during an insect's life cycle. A relevant factor is the occurrence of pathogens such as bacteria, viruses, fungi, and parasites that are responsible for about 80% of the diseases in insect populations^1^. Recent research has highlighted several cases of success in insect invasions facilitated by microorganisms^2–6^, including some microsporidia.

Microsporidia have been reported to cause substantial deleterious effects on host fitness in host insects. These effects include malformations in infected pupae, increased larval mortality, developmental delay of immatures, reduced fertility and longevity of adults, and increased susceptibility to stress conditions^7^. These stress factors cause biological changes in the host insect and may be associated with a decrease in its rate of parasitism^1^. As microsporidian pathogens generally display efficient transmission mechanisms and moderate virulence, these traits may make them more effective agents in establishing enzootics in host population^8^, as evidenced by the use of a microsporidium to control grasshoppers^9^. In this context, microsporidian entomopathogens hold a great potential as long-term biocontrol agents of numerous arthropod pests, but their natural incidence and pathogenicity in populations of forest pests have been underexplored^10^.

In the past, microsporidia were considered to be spore-forming protozoa, but in the light of modern taxonomy, this group was relocated to in or near the Fungi kingdom and are now called non-flagellate, single-celled fungi, and obligate intracellular parasites^11^. Several studies suggest a new classification for microsporidia within or near the fungi group, and the majority of entomopathogenic microsporidia belong to the genus *Nosema*, with more than 150 insect host species described in 12 insect orders, notably Lepidoptera, Hymenoptera, Diptera, Orthoptera and Coleoptera are among them^12^.

Microsporidia generally cause sublethal and chronic deleterious effects in infected hosts and, as a result, are a serious problem in insects that are massively rearing in laboratories and bio-factories^1,7^. Spores are generally small and most entomopathogenic microsporidia are approximately 2 to 6 µm in length. Spores can have different morphologies, including rounded, oval, or pyriform, and less frequently reniform, long ovals to almost tubular shape and refringent appearance when examined under phase contrast microscopy^7^.

The Eucalyptus snout beetle, *Gonipterus platensis* Marelli 1927 (Coleoptera: Curculionidae), also known as the eucalyptus beetle, is currently the primary coleoptereus pest in commercial eucalyptus forestry in Brazil. *Gonipterus platensis* is native to Australia, has a high destructive potential as adults and larvae feed mainly on young leaves^13^, and are distributed across the South and Southeast of Brazil^14^. In a study carried out in Portugal, defoliation by *G. platensis* resulted in wood losses of 648 million euros in the last twenty years^15^_._

Despite microsporidiosis being reported in a variety of different insect populations, there are no previous studies reporting the presence of this pathogen in populations of *G. platensis* in the literature. Recently, microsporidiosis symptoms observed in infected adult beetles (males and females) included typical morphological malformations comprising an abnormal abdomen with the second pair of wings displaced, which impairs flight activity in a laboratory colony. This promptly motivated us to identify the pathogen and determine its incidence in natural populations. Identifying the pathogen would also allow us to evaluate the potential of the organism to serve as a potential biological control agent, as it can both cause slow mortality and more pronounced reduction in host fitness and deformations in adults, or at least an ecological factor in a biological control program developed towards this insect pest in forest plantations. The knowledge of a microsporidium associated with *G. platensis* is of great importance to understand the ecology of this insect pathogen interactions. It may also contribute to the development of a conservative biological control program with this entomopathogen that can negatively impact on this pest population. This knowledge is also relevant to prevent the pathogen from infecting laboratory-grown or mass-reared colonies of this insect.

## Results

### Morphological characterization and spore density of microsporidium

**Figure 1:**
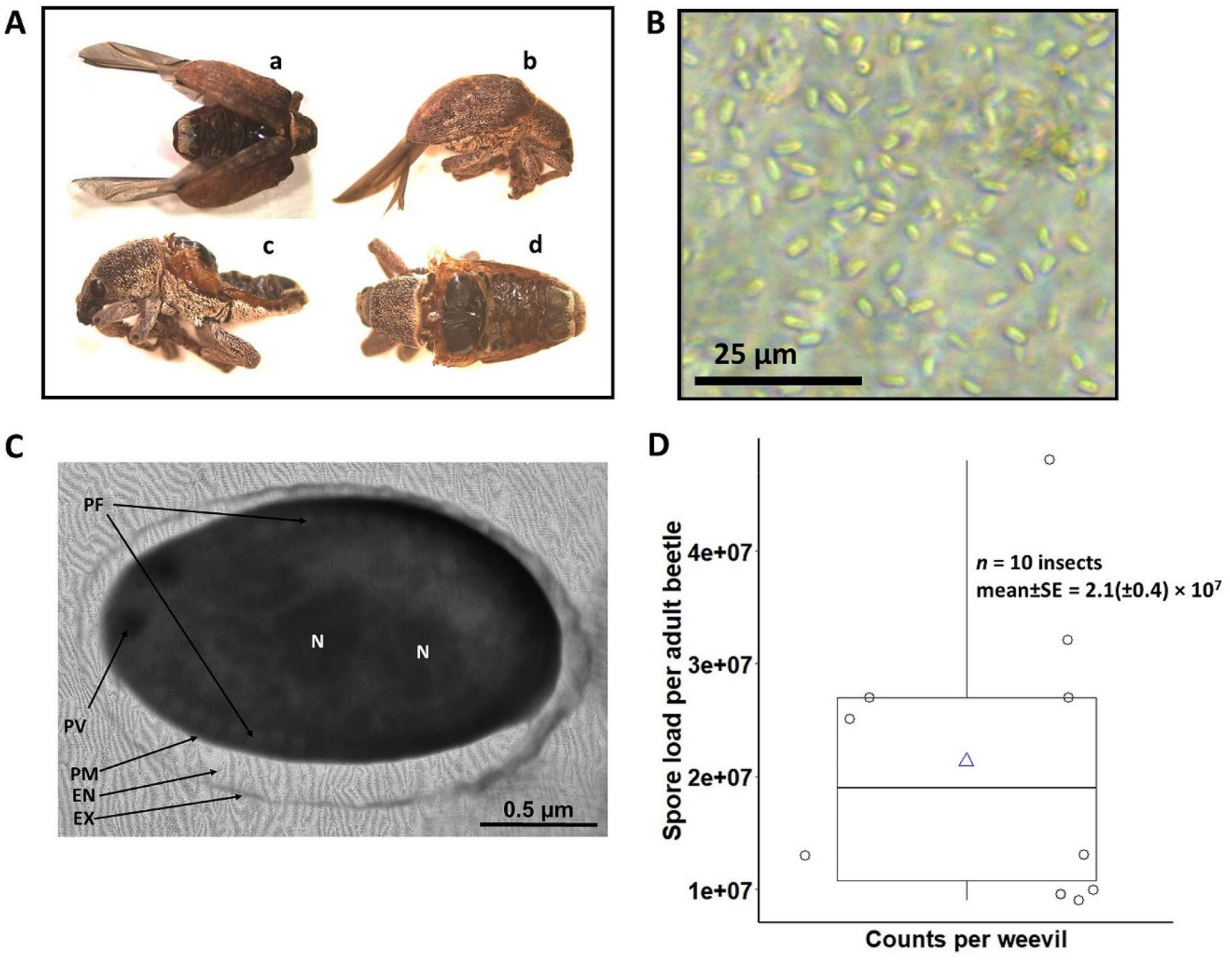
Disease diagnosis for the new microsporidiosis in symptomatic adult beetles and spore load in infected beetles. (**A**) Field-collected *Gonipterus platensis* adult beetles depicting visible symptoms of microsporidiosis disease. (**a**) Spread pair of wings exposing the abnormal and shrivelled abdomen; (**b**) Side view of a dead insect showing the membranous pair of wings extended from the elytra; (**c**) Wingless adult with abnormal abdomen. (**d**) Dorsal view of the abnormal abdomen. (**B**) Microsporidian spores found in the haemolymph of *G. platensis* adults, observed under a phase contrast microscope. Scale bar = 25 µm. (**C**) Electron micrograph of a longitudinal section of a mature microsporidian spore. The nucleus (binucleate, N), exospore (Ex), endospore (En), plasma membrane (PM), polar filament (PF) coiling posterolaterally around central diplokaryon showing 8 coils, and posterior vacuole (PV) are visible. Scale bar = 1 µm. (**D**) Density of spores per insect determined by enumerating the spore load from smeared symptomatic beetles and performing counts under a phase contrast microscope using a Neubauer chamber at × 400 magnification. (symbols on the boxplot: circle = data points, triangle = mean).

Field-collected *G. platensis* adult beetles were observed with visible symptoms of microsporidiosis disease, as they exhibited notable malformations characterized by spread pair of wings with abnormal and shrivelled abdomen, and the membranous pair of wings completely extended from the elytra (Fig. 1A). In this study, oval spores were identified as resistance structures of intracellular parasites isolated from the body content of *G. platensis* adults. In slides prepared with the beetle’s body content, a large amount of spores of the pathogen was easily and clearly observed, which was also used as a quick and easy diagnosis parameter to confirm microsporidiosis in field-collected beetles from different localities, as described later in this study (Fig. 1B). Microscopy is an inexpensive and routine technique for the diagnosis of microsporidiosis, but it is not an accurate method for species identification since the morphological structures of some pathogens are similar^16^.

Light microscopy revealed that fresh *Vairimorpha* sp. spore were generally elongated ovoid or oval shapes. Spore dimensions (n = 10) averaged 2.07 (± 0.136) µm in length and 1.20 (± 0.066) µm in width, respectively (Fig. 1B). Ultrastructure of microsporidian spores were examined under transmission microscopy and revealed spore wall consisted of an electron-dense exospore. The coiled region of the polar tube comprised 8 turns, and the diplokaryotic nuclei were slightly separated from each other (Fig. 1C). Yet, this does not discard that this pathogen also forms uninucleate spores as well. All the above-mentioned cellular features corresponded to the basic characteristics found in the genus *Vairimorpha*. Microsporidian spores from 10 beetles with similar size and weight were collected in *Eucalyptus* plantation located at São Jerônimo da Serra, SP, Brazil, and presented a sex ratio of 1:1. As result, the averaged of spore load of symptomatic beetles was determined at 2.15 (± 0.402) × 10^7^ spores per adult with (Fig. 1D).

### Molecular identification and phylogenetic construction

**Figure 2:**
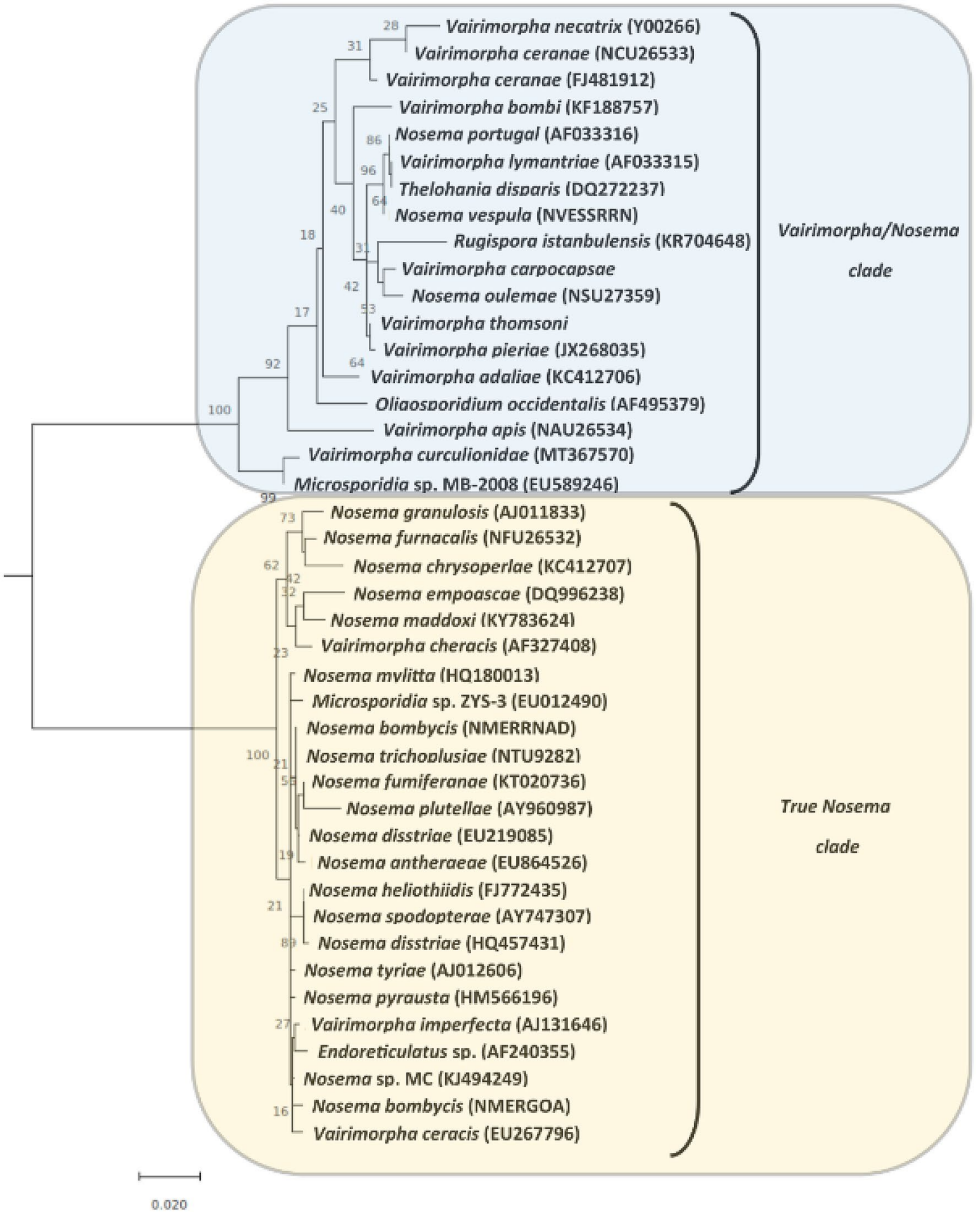
Phylogenetic tree based upon a small subunit rRNA gene alignment of 1068 positions in MEGA X with the Maximum Likelihood method using the Tamura 3-parameter model with a gamma distribution model. Values at branches indicate bootstrap support. The new microsporidium isolate is assigned to the *Vairimorpha* genus with a proposed name of *Vairimorpha curculionidae*. The outgroup was *Ordospora colligata* and is not shown.

Sequencing of the SSU rRNA gene was performed to confirm the identification of the pathogen found infecting populations of *G. platensis*. The PCR results confirmed the isolate was closely related to *Microsporidia* species, with the highest sequence identity (98%) to a sequence submitted to Genbank as *Microsporidia* sp*.* MB-2008 (GenBank accession no. EU589246). A phylogenetic analysis of the strain and closely related strains of *Microsporidia* species was conducted (Fig. 2). The closest phylogenetic neighbor of the isolate found in *G. platensis* was found to be *Microsporidia* sp. MB-2008, which was isolated from another weevil, *Otiothynchus sulcatura* (Coleoptera: Curculionidae), followed by *Vairimorpha apis,* isolated from *Apis cerana* (Hymenoptera: Apidae). Based on the recent formal redefinition of the genera *Nosema* and *Vairimorpha* (Microsporidia: Nosematidae), the strain belongs to the *Vairmorpha* genus. Based on these findings, the name *Vairimorpha curculionidae* is proposed, when the species is formally circumscribed in the future.

### Prevalence of microsporidiosis in different field populations of *Gonipterus* spp.

**Figure 3:**
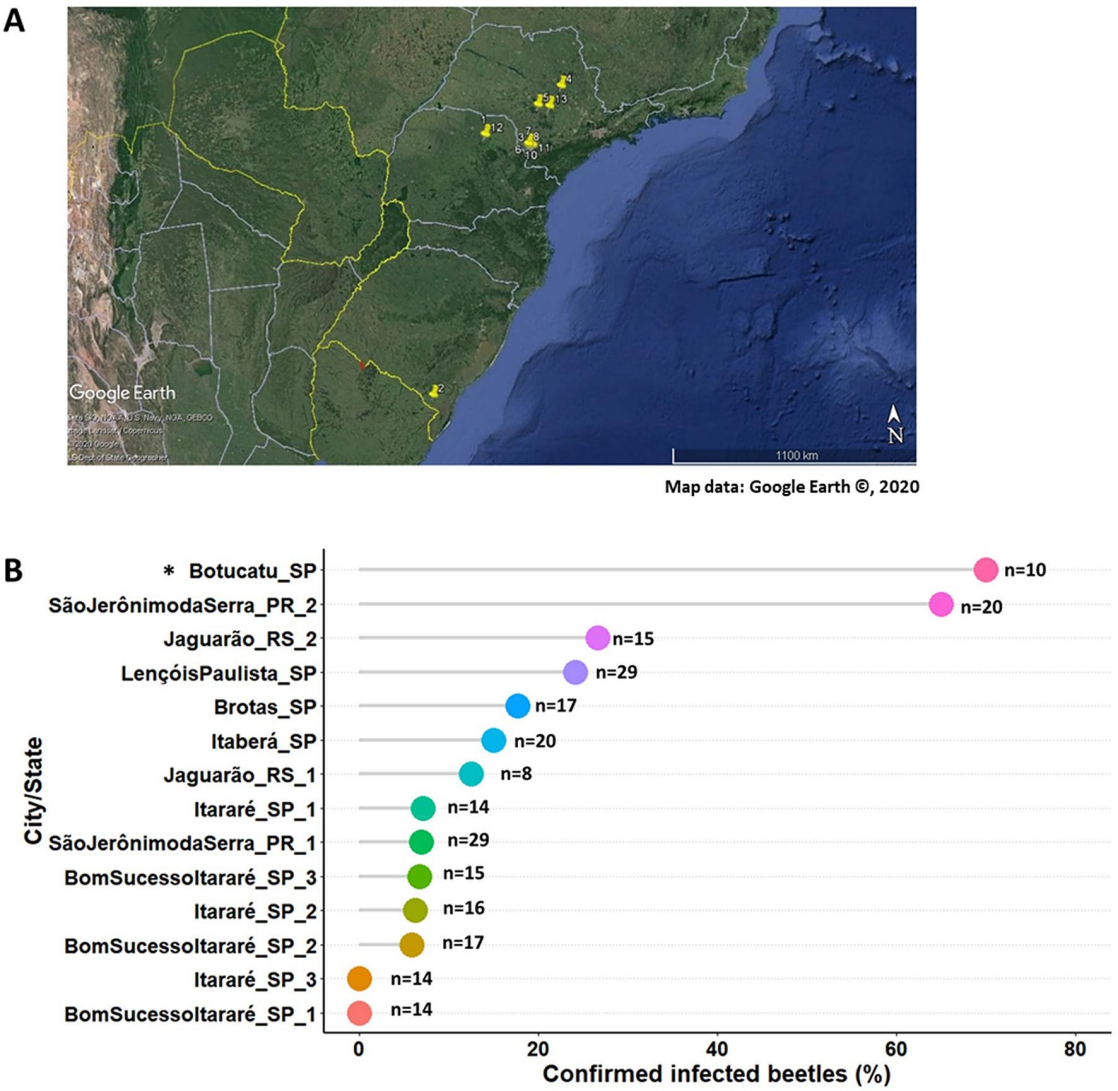
Natural prevalence of the new microsporidian pathogen in different field populations of the *Eucalyptus* snout beetle. (**A**) Collection field sites (marked with yellow pins) mapped with Google Earth showing where *Gonipterus platensis* adults were sampled in South and Southeast Brazil from commercial *Eucalyptus* plantations. (**B**) Proportion of infected beetles (number of infected versus number of healthy adults) by location. Survey of 14 distinct geographical points were performed to collect adult beetles (where n = number of total beetles collected in each site) for diagnosis based on typical microsporidiosis symptoms and presence of microsporidian spores in the haemolymph expressed as percent of confirmed infected beetles: one collection point was made in Botucatu, SP (*from insect colony kept in the lab); three samples from Bom Sucesso do Itararé, SP; three from Itararé, SP; one from Lençóis Paulista, SP; one from Brotas, SP; one from Itaberá, SP; two from Jaguarão, RS (*Gonipterus pulverulentus* was collected only in sample 2); and two from São Jerônimo da Serra, PR.

To determine the presence of microsporidium in other populations of the host insect, collections were made at 13 different sampling points distributed in three states as shown in the Fig. 3A. Identification of infected adult beetles was based on visual diagnosis of the microsporidiosis symptoms and by checking for spores in haemolymph. Positive results were obtained for microsporidium in most surveyed field sites, including beetles sampled from a second-generation, mass-reared laboratory insect colony in Botucatu-SP. There was a significant variation in natural infection caused by microsporidium across different regions or sites of collection in South and Southeast Brazil (*χ*^2^ = 62.87, df = 13, *P* < 0.0001). The prevalence of natural infection in these field-collected beetle populations sampled at different locations under field conditions ranged from 0 to 65%, but the highest pathogen incidence was observed in the insect colony maintained at the laboratory corresponding to 70% infection in adults confirmed by microscopic examination of the haemolymph sample, and these infected beetles were generally associated with typical symptoms of microsporidiosis previously described here (Fig. 3B). The percentage of mortality of beetles were not calculated in these field populations, because these insects were collected alive and them immediately frozen prior to taking to the laboratory. Interestingly, it was the first time that we found microsporidium infecting *Gonipterus pulverulentus* (26.7% infection), despite the lower frequency of this insect species compared to *G. platensis* throughout Brazil.

## Discussion

The present work is the first to describe the occurrence of microsporidiosis in an insect colony from the second generation and in field populations of *G. platensis* collected in commercial *Eucalyptus* plantations, with the disease being confirmed in two species present in Brazil, *G. platensis* and *G. pulverulentus* (Coleoptera: Curculionidae). Microsporidian infections have been reported in other Curculionidae^17,18^ as well as other coleopterans^19–22^, which demonstrate that this fungal pathogen is more common than previously thought infecting this diverse insect order. Adult beetles collected across the three Brazilian states that tested positive for the presence of this pathogen. Prevalence of natural infection in these field populations varied from 0 to 65% and 70% infection was detected in the laboratory-grown colony, indicating that this pathogen is spreading with expansion of the pest in Brazil and is established in mass-reared laboratory-grown colonies of *G. platensis*. However, additional research should still focus on the development of specific gene markers for rapid and accurate detection of this microsporidium in both asymptomatic and symptomatic *G. plantesis* beetles in complement to the traditional microscopic examination.

More than 1400 microsporidian species have been described so far and new species are being discovered each year^23,24^. There are several reports of these microorganisms infecting lepidopterans^25^, hymenopterans^26^ and orthopterans^27^, demonstrating the high genetic plasticity of this group of pathogens. Even more important is the frequent occurrence of microsporidian epizootics in laboratory colonies, in which there is high aggregation and population density of insects facilitating pathogen spread and new infections^28^.

Microsporidiosis is considered an important problem in the life cycle of insects^29^ because of the reduction in pupal size, number and viability, along with a longer duration of the pupal stage^30^. We also described conspicuous morphological abnormalities in infected *G. platensis* and *G. pulverulentus* beetles, and these symptoms are good indicators of microsporidiosis diagnostic alongside the presence of spores in the haemocoel. Additionally, spore numbers in *G. platensis* adults beetles reached an average concentration of 2.15 (± 0.40) × 10^7^ spores in symptomatic insects. This is similar to other microsporidian infections, *Nosema cerane* in honey bees yielded 1.15 × 10^7^ spores/bee at 18 days post-inoculation^31^. However, diet had a considerable effect on the spore load observed in honey bees^31^. This difference illustrates the specific interaction between microsporidium and its host insect in regard to spore density for the development of microsporidiosis.

The morphological similarity between microsporidian species, particularly based on spore measurements in isolation, makes identification to species difficult. Therefore, other methods are needed to confirm identification. Classification based on spore morphology can be difficult and inconsistent because some microsporidia have complex life cycles and form various types of spores. In some cases, different sporulation cycles occur at different stages of the host. Some species can also form different types of spores in the same host and sometimes in the same tissues^32^. Such evidence indicates high diversity of the spore dimension; hence, molecular analysis is essential in the identification of microsporidian species^16^.

The SSU rRNA sequence has been widely used as a molecular marker to estimate phylogenetic relationships between microsporidia, because it is a highly conserved gene^12^. However, this gene alone cannot be used to distinguish closely related species. This is a limitation of this gene for a more refined phylogenetic separation between species of this pathogen^33,34^. Nevertheless, it can be used in the taxonomic classification at the genus level^35,36^. In this case, the species is well resolved, even amongst members of the same genus. Future efforts are planned for whole-genome sequencing of this pathogen and then elucidate their key genes related to the infection process in *G. platensis*.

In general, the role of microsporidia as insect pathogens requires us to consider their ecological effects when developing a pest control program. This pathogen group could cause a chronic disease, which is debilitating to the host^37^. Moreover, the transmission of microsporidia takes place by one or more means, including the ingestion of spores present in the environment, and parental transmission to offspring, which facilitates their multiplication and persistence in the target population^38^. This high transmissibility of microsporidia in host population coupled with low lethality are key for their enzootic and long-term prevalence, which may not be a suitable feature for an applied biological control agent, but it could be a desirable for conservative biological control strategy towards forest pests. Despite the method of transmission in *G. platensis* is currently unknown. Furthermore, we have observed that infected symptomatic *G. platensis* beetles under laboratory conditions survive shorter and females do not lay eggs (*i.e.*, impaired fecundity) in comparison to healthy beetles (data not shown).

Due to their low virulence, microsporidia act slowly in host death and are likely insufficient to control an insect pest when used alone^39^. However, when used in combination with another type of chemical or microbiological insecticide, it reveals enormous potential and tends to be the most successful, as recently reported for the synergistic effect when combining *Metarhizium brunneum* with *Paranosema locustae* in the control of the migratory grasshopper *Melanoplus sanguinipes*^40^, as well as improved grasshopper control achieved by combined application of *P. locustae* and an insect growth regulator, flufenoxuron^41^. More importantly, there is a need to know in advance the environmental effects on the pathogen cycle in its host, in order to analyze the different strategies of release of the microsporidian pathogen in the field. In this sense, based on the historical use of microsporidia as biocontrol agents, especially in the case of *P. locustae* for management of locusts and grasshoppers, past lessons indicate that successful use of microsporidian pathogens for pest control is still a challenge in face of numerous drawbacks related to their production (they are obligatory pathogens), formulation, storage, and efficacy^42^. It also must be considered whether the microsporidium have a negative effect on control actions. It was recently reported that infection with a microsporidium reduced the efficacy of a granulovirus in larvae of *Phthorimaea* operculella^43^. While infection with *Bacillus thuringiensis* made *Galleria mellonella* more susceptible to infection by a microsporidium^44^. The lack of information about the ecological and biological aspects in the host–pathogen interactions of this new microsporidium with *G. platensis* makes this obligatory parasite an important natural enemy of this insect pest. Accordingly, the current study provides the first insight on the interaction between the eucalyptus snout beetle and its unreported microsproridium with a focus on the pathogen incidence in field and lab-reared beetle populations, molecular and morphological characterization, and description of typical microsporidiosis symptoms associated with infected adults.

Associated pathogens may also be present in other countries, so there is a need for a more in-depth study aimed at detecting the microsporidium in Australia, the native range of *Gonipterus,* and in other countries where *G. platensis* and other species of *Gonipterus* are present. We also need to investigate the host spectrum of this new microsporidiosis to different *Gonipterus* species as well as to non-target native beetles in Brazil, especially concerning predatory beetles, in order to find out if this pathogen could be lethal or harmless to non-target hosts.

In summary, this data indicates a probable new species of this pathogen, providing support for new studies on its biology and distribution, as well as identifying its potential to be a positive or negative factor in forest protection programs against *Gonipterus* spp. in Brazilian eucalyptus plantations.

## Materials and methods

The research was conducted in the Laboratory of Biological Control of Forest Pests and Molecular Biology Laboratory in Department of Plant Protection, School of Agricultural Sciences, São Paulo State University (UNESP), Botucatu, SP, Brazil.

### Colony maintenance of *Gonipterus platensis*

Adults of *G. platensis,* collected during field surveys in *Eucalyptus* plantations located in Botucatu, SP, Brazil, were housed in wooden cages (40 × 45 × 80 cm) with glass roofs and sides covered with voile fabric. These adults were fed on young leaves and buds of *Eucalyptus urophylla* (clone 433) (which were cleaned with hypochlorite solution (0.01%) and neutral detergent prior to use), maintained in an air-conditioned room at 25 ± 1 °C, RH 50 ± 10% and photoperiod of 12:12 h light:dark.

### Genomic DNA extraction

For the extraction of genomic DNA, five insects with symptoms of infection (found dead with open wings and deformed abdomen) were washed in 0.85% (w/v) NaCl sterile solution (Fig. 1). To extract genomic DNA from *G. platensis*, the abdomens of the five adults were macerated and added to a microtube with 160 µL of 10% Chelex solution (Sigma-Aldrich) and 8 µL of 20 mg/mL proteinase K. The samples were placed in a thermal block at 95 °C for 20 min, following a protocol for DNA extraction^45^, with Chelex 100 resin (Sigma-Aldrich). The DNA was eluted and stored at − 20 °C until use.

### Sequencing of SSU rRNA

Polymerase chain reaction (PCR) was performed with Amplitaq Gold mastermix (Thermofisher Willington, MA) using the following parameters; 95 °C, 10 min, 35 cycles of 95 °C, 30 s; 58 °C, 30 s; 72 °C, 60 s. PCR primers targeting the SSU rRNA were: 18f, 5′-CACCAGGTTGATTCTGCC-3′ and 1537r, 5′-TTATGATCCTGCTAATGGTTC-3′^46^. The resultant amplicons were prepared using a Nextera XT library preparation kit and indices (Illumina inc, San Diego, CA). The samples were sequenced using an Illumina MiSeq system with a MiSeq V3 2 × 300 bp sequencing kit. The demultiplexed reads were quality trimmed to Q30 and assembled using CLC genomics workbench v20.0 (Qiagen inc., Valencia, CA). The consensus sequences for two full length SSU rRNA genes were accessioned in GenBank under MT367570-MT367571.

### Phylogenetic analysis

Phylogenetic analysis was conducted with MEGA X using Maximum Likelihood analysis and the Tamura 3-parameter model with a discrete gamma distribution (5 categories), as this model was found to be the best-fit using the maximum likelihood-based model selection algorithm implemented in MEGA X. The partial deletion (90%) option was used, and the level of bootstrap support was calculated from 1000 replicates.

### Determination of microsporidian spore density in beetles

Microsporidian spores were isolated from typically symptomatic insects, which were originated from the field, and maintained at the Laboratory for Biological Control of Forest Pests (LCBPF/UNESP, Botucatu, SP, Brazil). The infected insects were homogenized in nuclease-free water in 0.2-mL microtubes. The suspension was subjected to three series of centrifugation: 2000 rpm for 10 min followed by 2 cycles at 12,000 rpm for 1 min. After each centrifugation, the supernatant was discarded. The spores accumulated at the bottom of the tube forming a "pellet", which was later resuspended in nuclease-free water^7^. This procedure was performed individually for 10 insects, in order to determine the average concentration of spores per beetle (n = 10). The spores from each insect were purified and suspended in nuclease-free water and then immediately quantified with a Neubauer chamber at × 400 magnification under a phase-contrast microscope (Leica DM 2500, Leica Microsystems, Heerbrugg, Switzerland). At the end of the counts, the spore density per insect was determined.

### Phase contrast microscopy

Spore immobilization and photomicrographs were performed according to the methods described in Vávra and Maddox^28^. Fresh spores were visualized from macerated body contents of infected insects after dilution in sterile 0.85% NaCl solution (w/v). A drop of this macerate was transferred to a glass slide and the spores were examined under a phase-contrast microscope (Leica DM 2500, Leica Microsystems, Heerbrugg, Switzerland) at × 400 magnification.

### Transmission electron microscopy

The material was prepared at the Center for Electron Microscopy (Biosciences Institute—UNESP). Tissue samples from the digestive tract of *G. platensis* adults were fixed in Karnovsky's solution^47^ modified (glutaraldehyde 25% paraformoldehyde 8% and 0.2 M monosodium/disodium phosphate buffer solution, pH 7.3). The samples were cut into small fragments of up to 2 mm^3^ for better fixation and incubated for at least 3 h at room temperature. The samples were removed from the fixative and washed 3 times for 5 min in 0.1 M phosphate buffer with pH 7.3, followed by immersing the material in osmium tetroxide for 2 h. After, the material was washed 3 times for 10 min in distilled water and immersed in 0.5% uranyl acetate in distilled water for about 2 h, in order to have the block contrast, revealing/highlighting the nucleic acids. Dehydration was accomplished with a series of solution containing an increasing amount of acetone. The series consisted of 10 min in 50% acetone, 2 washes for 10 min in 70% acetone, 3 washes for 15 min in 90% acetone, and finally, 3 washes for 15 min in 100% acetone.

The infiltration was done slowly using a 1:1 mixture of Araldite resin + 100% acetone and left for approximately 12 h at room temperature. The fixed biological material was made in an appropriate form and further placed in an oven at 60 °C for 2 to 3 days. Semi-thin cuts (0.5 μm thick) were made for choosing the region of interest and selected regions were trimmed again to further reduce the block surface allowing ultrathin cuts (60–90 nm) to be made and placed in appropriate grids. The cuts were contrasted with a saturated solution of uranyl acetate in 50% alcohol for about 20 min, followed by lead citrate for 10 min.

The slides were firstly examined under a light microscope (Zeiss Axioscop 2, Germany) to select the materials to be subsequently observed in the electron microscope. The analysis of the material was performed using the transmission electron microscope JEM 1011 (JEOL, Inc., Peabody, MA), operating at 40.60, 80 and 100 kV JEO at the Electronic Microscopy Laboratory (ESALQ—USP, Piracicaba, SP, Brazil).

### Detection of microsporidiosis in different field-collected ESB populations from Southern Brazil

In order to confirm the presence of the microsporidian pathogen in different field populations from South to Southeast Brazil, insects from three different states were collected in commercial *Eucalyptus* plantations (Table 1). After collection in the field, the insects were brought to the laboratory, examined for microsporidiosis symptoms in adult beetles to calculate the percent natural infection, and then stored in a − 20 °C freezer prior to dissection and microscopy to confirm the presence of the pathogen.

**Table 1:** Field sampling of *Gonipterus* spp. beetles retrieved from different localities in South and Southeast Brazil in 2019 for assessment of microsporidian pathogen incidence.

Sample	City and State	Eucalyptus host	Geographical coordinates
1	São Jerônimo da Serra, PR	*E. grandis* × *E. urophylla*	− 23.7516S/− 50.7955W
2.1*	Jaguarão, RS	*E. dunnii*	− 32.3760S/− 53.3263W
2.2	Jaguarão, RS	*E. dunnii*	− 32.3760S/− 53.3263W
3	Itaberá, SP	*E. grandis* × *E. urophylla*	− 24.1403S/− 49.1091W
4	Brotas, SP	*E. grandis* × *E. urophylla*	− 22.2117S/− 47.9985W
5	Lençóis Paulista, SP	*E. grandis* × *E. urophylla*	− 22.8249S/− 48.8533W
6	Itararé, SP	*E. grandis* × *E. urophylla*	− 24.1958S/− 49.2242W
7	Itararé, SP	*E. grandis*	− 24.1768S/− 49.1959W
8	Itararé, SP	*E. grandis* × *E. urophylla*	− 24.1175S/− 49.2479W
9	Bom Sucesso do Itararé, SP	*E. grandis* × *E. urophylla*	− 24.1495S/− 49.0802W
10	Bom Sucesso do Itararé, SP	*E. grandis*	− 24.1484S/− 49.1054W
11	Bom Sucesso do Itararé, SP	*E. grandis*	− 24.1485S/− 49.0980W
12	Laboratory rearing (Botucatu, SP)	*E. grandis* × *E. urophylla*	− 22.8456S/− 48.4348W
13	São Jerônimo da Serra, PR	*E. grandis* × *E. urophylla*	− 23.7556S/− 50.7965W

Asterisk (*) indicates another species found during field surveys that was identified as *Gonipterus pulverulentus.*
